# Extract interaction detection methods from the biological literature

**DOI:** 10.1186/1471-2105-10-S1-S55

**Published:** 2009-01-30

**Authors:** Hongning Wang, Minlie Huang, Xiaoyan Zhu

**Affiliations:** 1 State Key Laboratory of Intelligent Technology and Systems, Tsinghua National Laboratory for Information Science and Technology, Department of Computer Science and Technology, Tsinghua University, Beijing 100084, PR China

## Abstract

**Background:**

Considerable efforts have been made to extract protein-protein interactions from the biological literature, but little work has been done on the extraction of interaction detection methods. It is crucial to annotate the detection methods in the literature, since different detection methods shed different degrees of reliability on the reported interactions. However, the diversity of method mentions in the literature makes the automatic extraction quite challenging.

**Results:**

In this article, we develop a generative topic model, the Correlated Method-Word model (*CMW *model) to extract the detection methods from the literature. In the *CMW *model, we formulate the correlation between the different methods and related words in a probabilistic framework in order to infer the potential methods from the given document. By applying the model on a corpus of 5319 full text documents annotated by the *MINT *and *IntAct *databases, we observe promising results, which outperform the best result reported in the *BioCreative II *challenge evaluation.

**Conclusion:**

From the promising experiment results, we can see that the *CMW *model overcomes the issues caused by the diversity in the method mentions and properly captures the in-depth correlations between the detection methods and related words. The performance outperforming the baseline methods confirms that the dependence assumptions of the model are reasonable and the model is competent for the practical processing.

## Background

### Interaction detection method extraction

The study of protein interactions is one of the most pressing biological problems. In the literature mining community, considerable efforts have been made to automatically extract the protein-protein interactions (*PPI*) from the literature [1-3] and some practical systems have been put into use [4,5].

Nevertheless, little work has been done to automatically extract the interaction detection methods from the literature. The detection methods available to identify protein interactions vary in their level of resolution and the confidence of reliability. Therefore, it is important to identify such detection methods in order to validate the reported interactions. Some interaction databases, such as *MINT *[6] and *IntAct *[7], require the interaction entries to be experimentally confirmed. However, manually annotating the detection methods in the literature is time-consuming: on average, the curation of a manuscript takes up 2–3 hours of an expert curator [8]. Therefore, there is great practical demand of automatically extracting the detection methods from the literature.

The first critical assessment of detection method extraction was carried out by the *BioCreative II *challenge evaluation [9]. But only two groups (out of sixteen) submitted their results.

The diversity of method mentions in the literature is the major obstacle precluding the automatic extraction. In the real situation, different authors prefer different words and phrases to describe the same methods. For example, the detection method "*two hybrid*" (MI:0018) has 7 related synonyms, e.g. "*2-hybrid*", "*2 H *", "*2 h*", "*classical two hybrid*", "*Gal4 transcription regeneration*", "*two-hybrid*", "*yeast two hybrid*", and one exact synonym, e.g. "*2 hybrid*", in the MI ontology [10] definition (it includes the terms describing the interaction detection methods). Although the ontology has already included so many different descriptions, biologists would just mention "*yeast 2-h*", which is not included in the ontology, in their manuscripts.

To validate the diversity, we apply a string matching algorithm with all the names/synonyms from the MI ontology on a set of 740 documents, annotated with 96 methods and provided by the *BioCreative II *challenge evaluation. The matching performance is demonstrated in Table 1.

**Table 1:** String matching performance.

	*Precision*	*Recall*	*F-Score*
740 Full Texts	0.090	0.107	0.098

We apply a string matching algorithm with all the names/synonyms from the MI ontology on a set of 740 documents, annotated with 96 methods and provided by the *BioCreative II *challenge evaluation.

As Table 1 illustrates, the poor recall performance confirms the serious diversity, and the inferior precision stems from the simple matching algorithm, which does not take the context into consideration, since most of the matched names are not the exact methods applied in the document but the background knowledge. In this sense, the rigid dictionary-based matching strategy fails to address the practical problem.

Another straightforward solution is to treat the extraction issue as a classification problem – for each detection method in the ontology definition, a set of binary classifiers are built to make yes/no decisions [11,12]. But the traditional discriminative classifiers make little attempt to uncover the probabilistic structure and the correlation within both input and output spaces. In the biological domain, ignoring the correlation within both methods and words would hinder the performance since there are intrinsic relations.

In another point of view, from the perspective of involvement of domain experts, some approaches achieved acceptable results on the small data set. In Rinaldi's work [13], they invited the biologists to summarize the keywords and patterns for the extraction task and manually refined the patterns according to the performance. Obviously, this manner is not suitable for the large-scale data processing and its flexibility is not desirable.

### Generative topic model

Nowadays, in the machine learning community, the generative topic model is receiving more and more attentions. Latent Dirichlet Allocation (*LDA*) [14] is one of the most typical models. *LDA *reduces the complex process of producing a document into a small number of simple probabilistic steps and thus specifies a probability distribution over all possible documents. Using standard statistical techniques, one can invert the process and infer the set of latent topics responsible for generating a given set of documents [15].

*LDA*-like topic models are rapidly developed into quite different domains. Xing Wei [16] introduced the *LDA *model into information retrieval system and improved the retrieval performance; David Mimno [17] proposed the Author-Persona-Topic model to formulate the expertise of authors based on their publications; Fei-Fei Li [18] advanced a hierarchical generative model to classify natural scene in an unsupervised manner.

The advantages of the generative topic models are: 1) it would be easy to postulate complex latent structures responsible for a set of observations; 2) the correlation between different factors could be easily exploited by introducing the latent topic variables.

In this article, in order to extract the detection methods from the biological literature, we propose to formulate the correlation between the detection methods and related word occurrences in a probabilistic framework. In particular, we assume the applied methods are governed by a set of latent topics and the corresponding word descriptions are also influenced by the same topic factors, which characterize the correlation between the methods and related words. Under this setting, we appeal to the generative topic model to capture such latent correlations and infer the potential methods from the observed words by the statistic inference technique.

The intuitive notion behind the proposed model is that: different documents contain informative commonality in the descriptions of the same methods, therefore we propose to discover the common usage patterns for the desired methods from the latent correlations between the methods and related words. This manner is somehow analogous to the idea that to extract templates from the overlapping of different method descriptions. But the diversity in the method mentions brings the traditional template generation algorithms with low support and low confidence problems. Furthermore, when there are multiple methods in one document, the traditional approach would fail to figure out the latent correlations. In contrast, the generative model deals naturally with the missing data and provides a more feasible and theoretical framework.

The paper is organized as follows: in the Methods section, we present detailed descriptions about the proposed model and discuss the inference and parameter estimation procedures for the model; in the Results section, we perform extensive experiments to validate the proposed model; and in the Conclusions section, we would conclude the work and demonstrate our contributions in this paper.

## Methods

### Correlated Method-Word model

**Figure 1:**
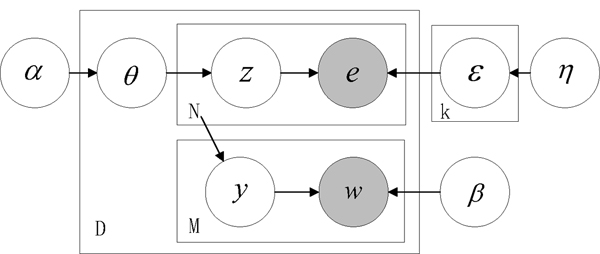
**Graphical model representation of the CMW model**. Following the standard graphical model formalism [19]: nodes represent the random variables and edges indicate the possible dependence. The joint probability can be obtained from the graph by taking the product of the conditional distribution of nodes given their parents.

We present the Correlated Method-Word model (*CMW *model) to extract the detection methods from the biological literature. The *CMW *model is depicted in Figure 1 with graphical representation. In the standard graphical model formalism [19]: nodes represent the random variables and edges indicate the possible dependence. The joint probability can be obtained from the graph by taking the product of the conditional distribution of nodes given their parents, see Eq(1).

The model can be viewed in the terms of generative process that, the author should first select a set of topics for his/her manuscripts (e.g. physical protein-protein interactions); under different kind of topics, there are different choices of detection methods to confirm the findings (e.g. *pull down *to confirm protein interactions); the selected methods are represented by the particular word occurrences (e.g. descriptions of the experiment conditions, properties and materials), which are also governed by the selected topics. Therefore, the correlations between the detection methods and related words are characterized by the latent topic factors; and from the observed words, we are able to infer the potential methods in the given document according to such correlations.

Formally, we define a corpus consists of *D *documents, *E *methods and *V *words, and a given document consists of *N *methods and *M *words. To simplify the model, we have assumed the topic size *k *is known and fixed on the whole corpus. In the given document *d*, we denote *θ *as the document-specific topic distribution; **z **= {*z*_1_, *z*_2_, *z*_3_,..., *z*_*N*_} as the particular discrete topic assignments for each method; **y **= {*y*_1_, *y*_2_, *y*_3_,..., *y*_*M*_} as the indexing variables to indicate which topic factor generates the corresponding word and *ϵ *as the method distribution under the topics. These are the latent variables. *e *= {*e*_1_, *e*_2_, *e*_3_,..., *e*_*N*_} and *w *= {*w*_1_, *w*_2_, *w*_3_,..., *w*_*M*_} are the observed methods and words in document *d*. Besides, *α *and *η *are the parameters of *k*-dimensional and *E*-dimensional Dirichlet distributions that postulate the topic and method prior distributions on the corpus and *β *is a *k *× *V *matrix, which represents the word distribution under topics. These are the model parameters.

Conditioned on the model parameters (*α*, *β*, *η*), the *CMW *model assumes the following generative process of the methods and related words in one document:

1. Sample topic proportion *θ *from the Dirichlet distribution: *θ *~*Dir*(*α*)

2. For each method *e*_*n*_, n ∈ {1, 2, 3,..., N}:

a. Sample topic factor *z*_*n *_from the multinomial distribution : *z*_*n *_~*Mul*(*θ*)

b. Sample method *e*_*n *_from the multinomial distribution conditioned on *z*_*n *_: *e*_*n *_~*p*(*e*_*n*_|*ϵ*, *z*_*n*_)

3. For each related word *w*_*m*_, m ∈ {1, 2, 3,..., M}:

a. Sample indexing variable *y*_*m *_from the Uniform distribution conditioned on N: *y*_*m *_~*Unif *(1, 2, 3,..., *N*)

b. Sample word *w*_*m *_from the multinomial distribution conditioned on $z_{y_{m}}$: *w*_*m *_~*p*(*w*_*m*_|*β*, $z_{y_{m}}$)

Our basic notion about each component of this model is that, the discrete occurrences of detection methods and related words in the given document are governed by the topic-specific distributions (e.g. matrix *ϵ *and *β*) respectively. We use such conditional distribution to bridge the correlation between the methods and word occurrences: under different topics, there are different choices of detection methods and the corresponding word descriptions. To formulate this notion in a probabilistic framework, we follow the general settings in the *LDA *model that we assume the document-specific topic proportion *θ *is drawn from the *k*-dimensional Dirichlet distribution *Dir*(*α*), which determines the topic mixture proportion. Especially, we treat the parameter of method's multinomial distribution *ϵ *as a *k *× *E *matrix (one row represents for each mixture component), and, to avoid over-fitting caused by the unbalanced and sparse method occurrences, we assume that each row of *ϵ *is independently drawn from the *E*-dimensional Dirichlet distribution: *ϵ*_*i *_~*Dir*(*η*), which smooths the method distribution under each topic. Each row of matrix *β *represents the particular word distribution under the topics. Besides, since the correlation between the methods and word occurrences is underlying (a document usually associates with multiple detection methods), we use the indexing variable **y **to indicate such latent structure between them.

Thus, the joint probability on the observed methods, words and latent variables in one document is given as follows:

(1) $$p(e,w,\theta ,\epsilon ,y,z|\alpha ,\beta ,\eta )=p(\theta |\alpha )\prod_{i=1}^{k}p(\epsilon_{i}|\eta )\left(\prod_{n=1}^{N}p(z_{n}|\theta )p(e_{n}|\epsilon ,z_{n})\right)\left(\prod_{m=1}^{M}p(y_{m}|N)p(w_{m}|\beta ,z_{ym})\right)$$

**Figure 2:**
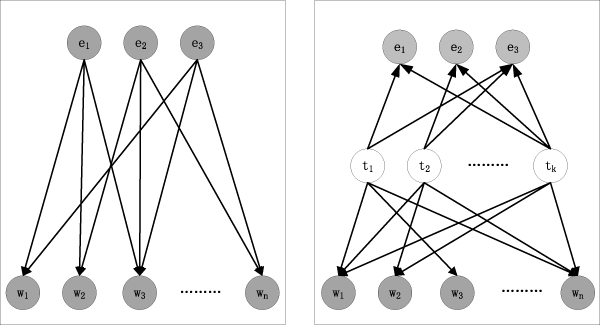
**Comparison between the traditional approach and the CMW model**. In the this representation, *e *denotes the detection methods associating with the document, *w *denotes the observed words and *t *in the right panel denotes the latent topic factors in the *CMW *model.

An intuitive comparison between the traditional approach (e.g. discriminative classification and template matching method) and the proposed *CMW *model is illustrated in Figure 2.

The traditional approach (the left panel of Figure 2) simply assumes the relation between the detection methods and related words is determined by the direct mapping. On the contrary, the *CMW *model (the right panel of Figure 2) formulates the relationship within a more throughout consideration: via the latent topic factors, word occurrences are formulated as a finite mixture under particular methods, so that they are not restricted to any methods and multiple words could contribute to the same method. This framework is more suitable and robust to deal with the diversity in the method descriptions. Furthermore, the discriminative classification algorithms assume the methods are independent in prior and the words are also independent when observing the given methods. Thus they would neglect the latent patterns within both methods and words. But in the *CMW *model, different topics govern dissimilar methods and words occurrences, embedding the correlation not only between different methods but also within the related words (see the Correlation between methods and words section and the Methods correlation analysis section for the detailed experiment results).

Efficient dimensional decomposition is explicitly implemented: *V*-dimensional word space and *E*-dimensional method space are mapped into the *k*-dimensional topic space, in which it will be easier for us to reveal the latent correlations between the detection methods and the variant word occurrences.

### Inference and parameter estimation

#### Variational inference

In order to utilize the *CMW *model, we need to compute the posterior distribution of the methods in a given document, that is:

$$p(e,\theta ,\epsilon ,y,z|w,\alpha ,\beta ,\eta )=\frac{p(e,w,\theta ,\epsilon ,y,z|\alpha ,\beta ,\eta )}{p(w|\alpha ,\beta ,\eta )}$$

Unfortunately, this posterior distribution is intractable: the couples between the continuous variable *θ *and discrete variable *β*, *ϵ *induce a combinatorial number of terms, making it impossible to efficiently get the exact inference result.

Although the exact inference is intractable, there are a wide variety of approximate inference algorithms can serve for the propose, including: expectation propagation [20], variational inference [21] and Markov chain Monte Carlo (*MCMC*) [22] etc. For computational efficiency, we develop a variational inference procedure to approximate the lower bound of the desired posterior distribution of methods in a given document.

In particular, we define the following fully factorized distribution on the latent variables:

(2) $$p(\theta ,\epsilon ,y,z|\gamma ,\phi ,\lambda ,\sigma )=p(\theta |\gamma )\prod_{i=1}^{k}q(\epsilon_{i}|\sigma_{i})\prod_{n=1}^{N}q(z_{n}|\phi_{n})\prod_{M=1}^{M}q(y_{m}|\lambda_{m})$$

where the Dirichlet parameters *γ*, *σ *and the Multinomial parameters *ϕ*, *λ *are free variational parameters.

The meaning of the above variational distribution is that: we discard the dependence among the latent variables by assuming they are independently drawn from the respective distributions. In that case, the aim of the variational inference is to find the optimal variational parameters which could minimize the *Kullback-Leibler *(*KL*) divergence between the variational distribution and the true posterior distribution.

Following the general recipe for the variational approximation, we take derivatives with respect to the variational parameters and obtain the following coordinate ascent algorithm:

1. Dirichlet parameter *γ*:

(3) $$\gamma_{i}=\alpha_{i}+\sum_{n=1}^{N}\phi_{ni}$$

2. Multinomial parameter *ϕ*:

(4) $$\log\phi_{ni}\propto \sum_{m=1}^{M}\lambda_{mn}w_{m}^{s}\beta_{is}+[\psi (\gamma_{i})-\psi (\sum_{n=1}^{k}\gamma_{t})]+e_{n}^{j}[\psi (\sigma_{ij})-\psi (\sum_{t=1}^{E}\sigma_{it})]$$

where $w_{m}^{s}$ means the *m*th word in the document is the *s*th one in the vocabulary, and $e_{n}^{j}$ means the *n*th method in the document is the *j*th method in the list.

3. Multinomial parameter *λ*:

(5) $$\log\lambda_{mn}\propto \sum_{i=1}^{k}\phi_{ni}w_{m}^{s}\beta_{is}$$

4. Dirichlet parameter *σ*:

(6) $$\sigma_{ij}=n_{j}+\sum_{d=1}^{D}\sum_{n=1}^{N_{d}}\phi_{dni}e_{dn}^{j}$$

These update equations are invoked repeatedly until the relative change in *KL *is small (< 0.0001%).

When we have achieved the approximate posterior probability, we could handle the conditional distribution of interest – *p*(**e**|**w ***α*, *β*, *η*) to infer the potential methods in a given document as follows:

(7) $$p(e|w,\alpha ,\beta ,\eta )\approx \sum_{n=1}^{N}\sum_{z_{n}}q(z_{n}|\phi_{n})p(e|\epsilon ,z_{n})p(\epsilon |\eta )$$

#### Parameter estimation

Following the similar procedure in the variational inference, in this section, we utilize an empirical Bayesian method to estimate the parameters of the *CMW *model. This time, we are looking for the optimal model parameters to tighten the lower bound of likelihood and obtain the following update equations:

1. Update the Dirichlet parameter *α *by the Newton-Raphson algorithm:

(8) $$\frac{\partial L(\alpha )}{\partial \alpha_{i}}=\sum_{d=1}^{D}\left\{\psi (\sum_{t=1}^{k}\alpha_{t})-\psi (\alpha_{i})+\psi (\gamma_{di})-\psi (\sum_{t=1}^{k}\gamma_{dt})\right\}$$

(9) $$\frac{\partial^{2}L(\alpha )}{\partial \alpha_{i}\partial \alpha_{j}}=D\left\{\psi^{\prime }(\sum_{t=1}^{k}\alpha_{t})-\delta (i,j)\psi^{\prime }(\alpha_{i})\right\}$$

where *δ *(*i, j*) = 1 when *j *= *k*, otherwise 0.

2. Update the Dirichlet parameter *η *by the Newton-Raphson algorithm:

(10) $$\frac{\partial L(\eta )}{\partial \eta_{j}}=\sum_{i=1}^{k}\left\{\psi (\sum_{t=1}^{E}\eta_{t})-\psi (\eta_{j})+\psi (\sigma_{ij})-\psi (\sum_{t=1}^{E}\sigma_{it})\right\}$$

(11) $$\frac{\partial^{2}L(\eta )}{\partial \eta_{i}\partial \eta_{j}}=k\left\{\psi^{\prime }(\sum_{t=1}^{E}\eta_{t})-\delta (i,j)\psi^{\prime }(\eta_{i})\right\}$$

3. Update the Multinomial parameter *β*:

(12) $$\beta_{js}\propto \sum_{d=1}^{D}\sum_{n=1}^{N_{d}}\sum_{m=1}^{M_{d}}\lambda_{dmn}w_{dm}^{s}\phi_{dnj}$$

These update equations correspond to find the maximum likelihood estimation with the expected sufficient statistics for each document taken under the variational posterior.

We develop an alternating *EM *procedure to find the optimal parameters as follows:

1. (*E-Step*) For each document in the training corpus, optimizing the variational parameters (*γ*, *ϕ*, *λ*, *σ*) according to equations (3) – (6);

2. (*M-Step*) Maximizing the resulting lower bound on the variational likelihood on the whole corpus with respect to the model parameters (*α*, *β*, *η*) according to equations (8) – (12).

The *E-Step *and *M-Step *are repeated until the bound on the likelihood converges (relative change in likelihood is less than 0.001%). The convergency rate of the process depends on the size of parameters in the model, (e.g. number of words, methods and topics). In our experiments (3000 words, 115 methods and up to 500 topics), the algorithm terminates in less than 30 iterations in all the cases.

## Results and discussion

We collect 5319 full-text documents from *PubMed *[23] with method annotations from another two public curated interaction databases: *MINT *and *IntAct*. We perform the following pre-processions on the data set: 1) parsing the HTML file; 2) converting the words into lower cases; 3) removing a standard list of 400 stop words, punctuations, and the terms occur less than 50 times; 4) stemming the words to its root by *Porter Stemming *[24]. We utilize the *macro-precision*, *macro-recall *and *macro-Fscore *[25] to evaluate the performance in average.

### Test corpora

**Figure 3:**
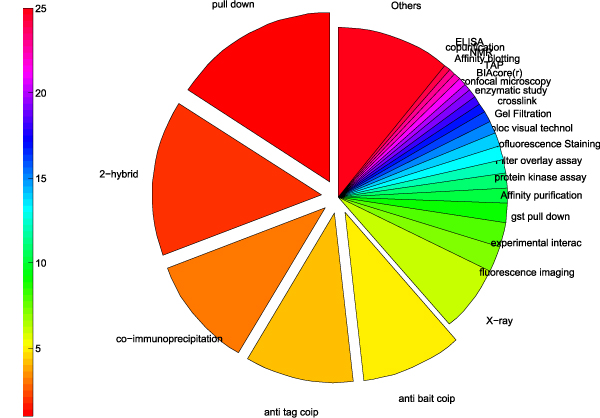
**Statistics of the corpus**. In the whole corpus, 5 dominate detection methods take up nearly 59.3% occurrences and 86.1% (99 out of 115) methods occur in less than 10% documents.

The whole corpus consists of 115 unique method annotations, and each document associates with 1.99 different methods in average. Unfortunately, the standard deviation of the method frequency is so large that the corpus is heavily unbalanced: the most popular method "*pull down*" (MI:0096) occurs 2040 times while there are 57 methods (49.6% of all) occurs less than 10 times. Figure 3 demonstrates the unbalanced method distribution on the whole corpus.

We can discover from Figure 3: 1) the 5 dominate detection methods, i.e. *pull down *(MI:0096), *2 hybrid *(MI:0018), *coip *(MI:0019), *anti tag coip *(MI:0007) and *anti bait coip *(MI:0006), take up nearly 59.3% occurrences in the whole corpus; 2) 86.1% (99 out of 115) methods occur in less than 10% documents. In this case, smoothing the estimated parameters is essential to achieve better performance.

### Feature selection

The *CMW *model is proposed to capture the correlation between methods and the "related" words. However, no curations explicitly annotate which words or sentences are related to the curated methods. So we employ *χ*^2 ^statistic [26] to select the most relevant feature words from the whole text.

Word *t*'s *χ*^2 ^value associating with the method *e *is calculated according to the following equation:

$$\chi^{2}(t,e)=\frac{N\times {\left(AD-BC\right)}^{2}}{\left(A+C)\times (B+D)\times (A+B)\times (C+D\right)}$$

where *A *is the number of times *t *co-occurs with *e*, *B *is the number of times *t *occurs without *e*, *C *is the number of times *e *occurs without *t*, *D *is the number of times neither *e *or *t *occurs, and *N *is the total number of documents.

By *χ*^2 ^statistic, we approximate the dependence between word *t *and method *e*, so that we can preserve the words, which are the most relevant to the method descriptions, by the following formulation:

(13) $$\chi_{avg}^{2}(t)=\sum_{i=1}^{E}p(e_{i})\chi^{2}(t,e_{i})$$

where *p*(*e*_*i*_) is the prior probability of method *e*_*i*_.

In the following experiments, we select the top 3000 terms to build up the feature set according to Eq(13).

### Effect of topic factors

We first use the perplexity as the criterion to evaluate the effect of the number of topic factors, which is the only arbitrary parameter in the *CMW *model. The perplexity on a set of testing documents is calculated as follows:

$$perplexity=\exp\{\frac{-\sum_{d=1}^{D}\sum_{n=1}^{N_{d}}\log p(e_{n}|w_{d})}{\sum_{d=1}^{D}N_{d}}\}$$

where *D *is the set of testing documents and *N*_*d *_is the number of methods in the document *d*.

Better generalization capability is indicated by a lower perplexity over the held-out testing samples. We held out 20% of collection for the testing purpose and used the remaining 80% to train the model, in accordance with 5-fold cross-validation.

**Figure 4:**
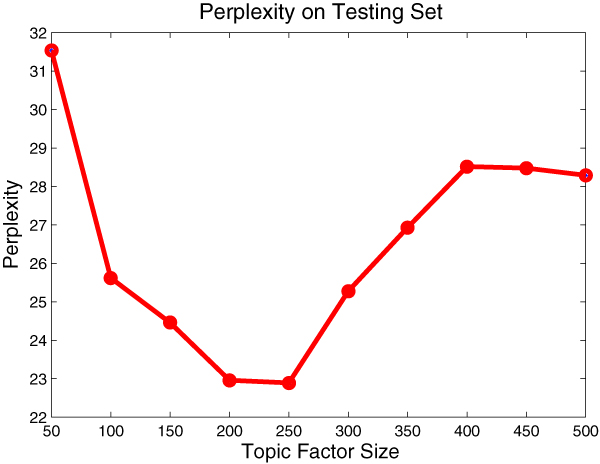
**Methods perplexity**. Lower perplexity on the testing data indicates a better generalization capability. Here we held out 20% of collection for the testing purpose and used the remaining 80% to train the model, in accordance with 5-fold cross-validation.

Figure 4 demonstrates that the generalization power of the *CMW *model gets improved with more topic factors. Since with more topic factors the documents could be partitioned into finer segments, more precise correlations between the methods and words could be captured. But as the number of topics exceeds a limit, the model becomes too specific (higher perplexity). Therefore we could conclude that the topic factors could be treated as the discriminate granularity of the model, that is it operates as a tradeoff between the generality and specificity. Besides, as the number of topic factors increase, there will be more parameters to be estimated (linearly increase with the number of topics), so that more training data is needed to obtain the reliable parameters. In this sense, when the number of topic factors exceeds a limit, the quality of the estimated parameters decreases and hampers the prediction power.

Besides understanding the impact of the number of topic factors on the generalization capability, we would be more interested in their explicit effect on the extraction performance. Here, we evaluate the precision and recall performance of the model under different number of topic factors. We use the same data set partition as in Figure 4.

**Figure 5:**
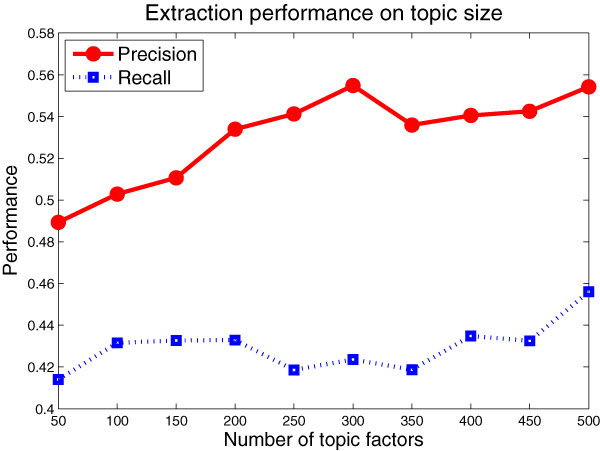
**Performance on the number of topics**. We use the same data set partition as in Figure 4 and evaluate the *precision *and *recall *performance of the *CMW *model.

We could discover from Figure 5 that the extraction performance peaks close to the place where the perplexity reaches the minimum. This is consistent with the foregoing perplexity result. These results give us insight about determining the proper size of topics for the *CMW *model.

### Extraction performance

Since there is few work to compare with, we employ the well studied *Naïve Bayes*, *KNN *and *SVM *as the baseline methods to evaluate the capability of the proposed *CMW *model. We choose *Naïve Bayes *because it is the simplest generative model with complete independence assumptions, and *KNN *model could exploit the heterogeneity among the similar documents. These are the two basic notions in the *CMW *model. Besides, *SVM *model is the most powerful discriminative model for the classification task with decent performance [11]. All the baseline models are operating on the same feature set as the *CMW *model employs.

In the *Naïve Bayes *model, we estimate the posterior probability of the methods in a given document by Eq(14). We use a pre-estimated threshold to retrieval the most probable methods.

(14) $$p(e|w)\propto \prod_{n}p(w_{n}|e)p(e)$$

In the *KNN *model, we make the prediction by ranking the candidate methods in the union of the unlabeled sample's *k*-nearest labeled neighbors, and weight the candidate methods by the similarity between the desired unlabeled sample and its neighbors.

In the *SVM *model, we follow Boutell's strategy [12] to train a set of binary classifiers for each method and predict the unknown methods by the classifiers' output. We use *SV M*^*light *^[27] toolkit to implement a linear kernel *SVM *model with the default parameters.

We perform comparisons on different proportions of the data used for training. In this comparison, we set the size of topics in the *CMW *model to be 250 and *k *in the *KNN *model to be 37.

**Figure 6:**
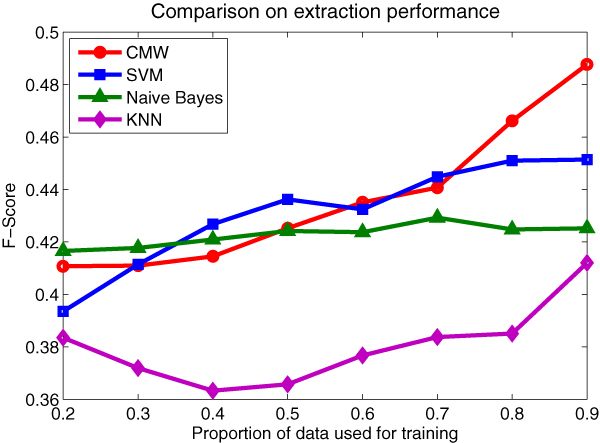
**Comparison with the baseline models**. We compare the *F-score *performance of the four models on different proportions of the data used for training. In this comparison, we set the size of topics in the *CMW *model to be 250 and *k *in the *KNN *model to be 37.

We could discover from Figure 6 that, as the training set increases, the performance of the *CMW *model improves rapidly. The reason for this phenomenon is that in the *CMW *model, there are *E *+ *k*(*V *+ 1) parameters to be estimated, when the training set is not large enough, most of the parameters cannot be fully estimated, which would directly hinder the performance of the model.

One thing we should note is that, since the data set is unbalanced, we should attend the retrieval performance on the minor methods as well. In the method-level evaluation, the baseline models only retrieve most of the major methods (e.g. the top 5 methods) but ignoring the other minor ones, while the *CMW *model exhibits superior retrieve power. We demonstrate the coverage performance of each model on the testing set to compare their retrieval capability.

**Figure 7:**
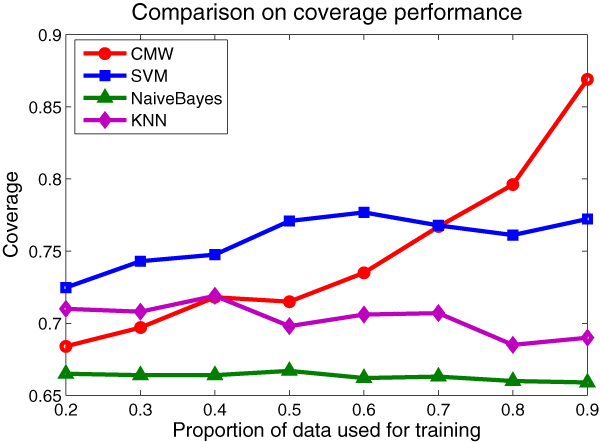
**Coverage comparison with the baseline models**. We compare the *coverage *performance of the four models on the same data set partition as in Figure 4 and we use the same model parameter settings.

Figure 7 demonstrates that the *CMW *model possesses better retrieval capability than all the baseline methods when the training set is large enough. We contribute the nice coverage performance to the smoothing factor introduced to the method distribution. Because the whole corpus is sparse and unbalanced, the minor methods possess little proportion in the training set. However, the baseline models do not take the sparseness into account, so that they fail to retrieve the minor ones from the testing cases. In contrast, the *CMW *model attends the smoothing issue and overcomes the sparseness.

Rinaldi utilized the expert revised patterns to perform the extraction and achieved the best performance in the *BioCreative II *challenge evaluation [13]. To compare with their approach, we operate the *CMW *model on the same testing corpus (300 full text documents) and set the topic size to be 300 according to the result in the previous section. The *CMW *model achieved competitive results (*F-Score *improved 12.4%), illustrated in Table 2.

**Table 2:** Comparison with BioCreative II best result.

	*Precision*	*Recall*	*F-Score*
*BioCreative II *Best Run	0.506	0.522	0.483

*CMW *model	**0.654**	**0.545**	**0.543**

*improvement*	**+29.2%**	**+4.4%**	**+12.4%**

We operate the *CMW *model on the *BioCreative II *testing corpus (300 full text documents) to compare with the best result reported in the evaluation. We set the topic size to be 300 according to the result in Figure 5. The *CMW *model achieved competitive results, *F-Score *improved 12.4%.

Here, we briefly conclude the performance of the *CMW *model. The extraction performance outperforms the discriminative baseline methods confirms that the dependence assumptions in the proposed *CMW *model are reasonable. Besides, the traditional discriminative classifiers fail to model the correlation within either the methods or the related words, while in the biological domain such correlations convey important domain dependent information. In this sense, the major advantage of the *CMW *model is that it properly exploits such informative correlations to reinforce the extraction performance. The improvements against the manually revised templates approach validate that the *CMW *model does exploit more precise and general patterns for the desired methods from the large-scale statistics, confirming the reasonable underlying semantic structure from another perspective.

### Correlation between methods and words

To demonstrate the correlation between the different methods and words exploited by the *CMW *model, we utilize the method-specific distribution over words by the conditional distribution *p*(*w*|*e*) to retrieval the most relevant terms under each desired method:

(15) $$s(w|e)=\frac{\sum_{d\in D}\log p(w|e)}{\sum_{d\in D}M_{d}}$$

where *D *is the set of documents associating with the desired method *e *and *M*_*d *_is the number of words in the document *d*.

In Table 3, we collect top 20 terms for 6 different methods according to Eq(15) from the corpus. We can see from the table, most of the terms are appropriately gathered to the given methods. For example, "*structure"*, "*crystal"*, "*helix" *are gathered to *x-ray*, and "*yeast"*, "*two-hybrid"*, "*site" *are gathered to *two hybrid*. These are the informative terms in the MI ontology definition of these methods. From this result, we could discover that the *CMW *model properly selects suitable "indicators" for the given methods. From another perspective, since these "indicators" are organized in a probability framework and accordingly contribute to the desired methods, the *CMW *model could better overcome the issue caused by the diversity in the method mentions. The reasonable word distribution under methods confirms that the *CMW *model captures the in-depth correlation between the methods and related words from the literature.

**Table 3:** Top 20 relevant terms for methods.

*Method*	*Terms*
**x-ray** (MI:0114)	structure, crystal, residue, molecule, model, site, form, interface, chain, contact, bond, hydrogen, helix, pp, record, helical, window, surface, linker, segment

**two hybrid** (MI:0018)	yeast, two-hybrid, interact, assay, fusion, system, plasmid, clone, cdna, screen, bait, sequence, acid, amino, encode, site, pp, record, domain, plant

**pull down** (MI:0096)	gst, fusion, glutathione, pull-down, assay, interact, bead, buffer, wash, yeast, scopus, min, incubate, two-hybrid, antibody, pp, record, system, plasmid, sequence

**anti tag coip** (MI:0007)	record, pp, cite, yeast, antibody, strain, panel, anti-flag, saccharomyces, flag, cerevisia, growth, blot, western, flag-tagg, gene, grow, medline, ha, anti-ha

**anti bait coip** (MI:0006)	control, buffer, pp, record, isi, bait, cancer, antibody, extract, c-terminus, bead, sirna, tumor, stain, gene, yeast, sds, luciferase, embo, cdna

**coip** (MI:0019)	antibody, pp, record, extract, yeast, domain, sequence, expression, blot, cdna, clone, activity, luciferase, growth, transfect, acid, fusion, sirna, mmedta, link

We collect top 20 terms for 6 different methods according to Eq(15) from the corpus.

### Methods correlation analysis

By the *CMW *model, we map different methods into the latent topic space, where we are able to analyze the relationship between the different methods. Meanwhile, there are intrinsic inherit relationships between the methods, defined in the MI ontology and organized as a concept hierarchy.

To represent a given method in the latent topic space, we re-normalize the topic-specific method distribution matrix *ϵ *by column as follows:

(16) $$r(e_{i})=\frac{\epsilon_{i}}{\sum_{s=1}^{k}\epsilon_{is}}$$

where *ϵ *_*i *_is the *i *th column of *ϵ *matrix.

Recall that, each row of the multinomial parameter *ϵ *is the method distribution under a particular topic, so that each column of *ϵ *represents a method in the topic space. By normalizing *ϵ *by column, we can represent the different methods over the latent topic factors.

Based on this representation, we employ an accumulative clustering algorithm to perform the hierarchical clustering and utilize a visualization tool *gCluto *[28] to demonstrate the captured "*pedigree*" tree. (We only illustrate part of the clustering result because of the page limit.)

**Figure 8:**
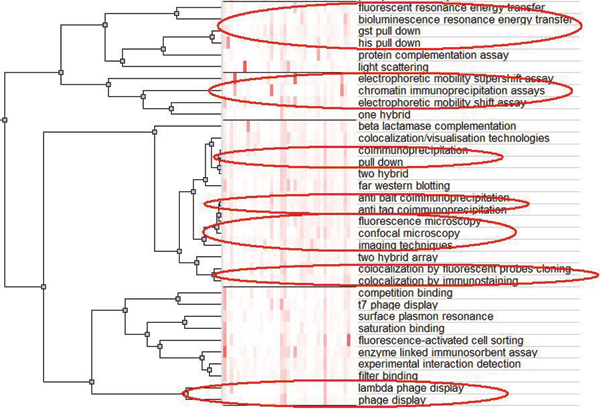
**Methods clustering tree**. We utilize an accumulative clustering algorithm to perform the hierarchical clustering and build up the "*pedigree*" tree of the detection methods. Red circles in the figure mean the correct clusters according to the MI ontology definition.

From the clustering result in Figure 8, we can discover that most of the sibling nodes within the MI ontology are successfully clustered with the correct hierarchy (red circles mean the correct clusters). The promising result confirms that the *CMW *model captures the proper correlations not only between the methods and words but also among the different methods. The traditional discriminative classifiers are not able to figure out such relationships.

### Classify irrelevant documents

Although the *CMW *model is proposed to address the extraction problem in documents with at least one detection method, in most situation, the curators don't know whether the document is *PPI *related or experimentally confirmed beforehand. So it is necessary to evaluate the model's capability to classify the irrelevant documents.

We randomly select 1000 documents from *PubMed*, none of which are annotated by *MINT *nor *IntAct*. These documents are taken as the irrelevant documents. Meanwhile, we randomly select another 1000 documents from the evaluation corpus as relevant documents. In Eq (17), we define the relevance score of each document by the posterior probability of the most potential method in that document as follows:

(17) $$relevance(d)=\underset{e}{\max}p(e|w_{d})$$

This measurement indicates the maximum probability of a document containing at least one interaction detection method.

**Figure 9:**
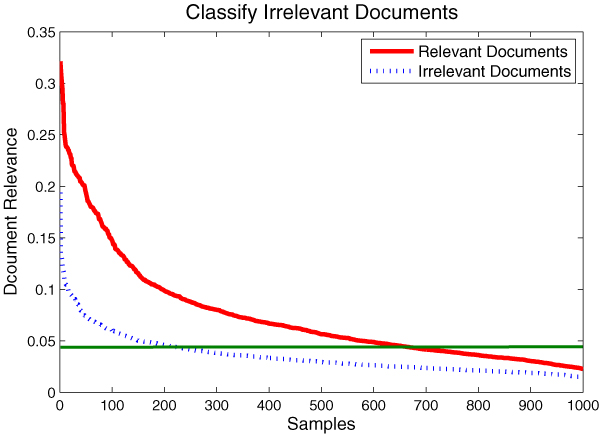
**Relevance distribution in relevant and irrelevant documents**. In the diagram, red line indicates the relevance scores in the relevant document set and the blue dots indicate the relevance scores in the irrelevant document set. If we select the classification threshold as the green line indicates, we would achieve a promising classification performance: in terms of precision 0.745, recall 0.676 and AUC 0.819.

We arrange the relevance scores in a descending order in Figure 9, so that it is easy to discover that the relevance scores in the relevant document set are significantly greater than those in the irrelevant document set. If we select the threshold as the green line indicated, we would achieve a promising classification performance: in terms of precision 0.745, recall 0.676 and AUC 0.819. The result indicates that the proposed *CMW *model possesses the capability to reject the irrelevant documents before extracting.

## Conclusion

In this paper, we propose a generative probabilistic model, the Correlated Method-Word model, to automatically extract the interaction detection methods from the biological literature. This problem is not well studied by the previous researches. By introducing the latent topic factors, the proposed model formulates the correlation between the detection methods and related words in a probabilistic framework in order to infer the potential methods from the observed words.

In our experiments, the proposed *CMW *model achieved competitive performance against the other well-studied discriminative classifiers on a corpus of 5319 full text documents. And it outperforms the best result reported in the *BioCreative II *challenge evaluation (*F-Score *improved 12.4%). From the promising results, we could see that the proposed *CMW *model overcomes the diversity in the method descriptions and appropriately solve the detection method extraction issue. Furthermore, the model captures the in-depth relationship not only between the methods and related words (see the Correlation between methods and words section), but also among the different methods (see the Methods correlation analysis section). Most of the discriminative classifiers fail to exploit such relations. The competitive performance confirms that the dependence assumptions in the model are reasonable and it is necessary to model the correlation between the different methods and words in the detection method extraction issue.

Our contributions in this paper lie in: 1) propose a generative probabilistic model with proper underlying semantics for the detection method extraction issue, and the model achieves promising performance; 2) properly model the correlation between the detection methods and related words in the biological literature, which captures the in-depth relationship not only between the methods and related words but also among the different methods.

The *CMW *model is now integrating to our *ONBIRES *system [5] to provide on-line service. And in the future work, we are planning to associate the extracted methods with the annotated interaction pairs and retrieve the evidence sentences in the documents, which would provide a more throughout annotation of the protein interactions in the biological literature.

## Competing interests

The authors declare that they have no competing interests.

## Authors' contributions

Wang carried out the major work of the paper, proposed the model, implemented the experiments and drafted the manuscript. Huang gave directions in the process and revised the draft. Zhu supervised the whole work, gave great amount of valuable suggestions and helped to revise the manuscript. All authors have read and approved the final manuscript.
